# Drivers' and conductors' views on the causes and ways of preventing workplace violence in the road passenger transport sector in Maputo City, Mozambique

**DOI:** 10.1186/1471-2458-11-800

**Published:** 2011-10-13

**Authors:** Maria T Couto, Per Tillgren, Maja Söderbäck

**Affiliations:** 1 Department of Public Health Sciences, Karolinska Institute, Norrbacka, 171 76, Stockholm, Sweden; 2 Faculty of Medicine, Eduardo Mondlane University, Av. Salvador Allende N°. 702, Maputo, Mozambique; 3 School of Health, Care and Social Welfare, Mälardalen University, 721 23 Västeras, Sweden

## Abstract

**Background:**

Workplace violence (WPV) is an occupational health hazard in both low and high income countries. To design WPV prevention programs, prior knowledge and understanding of conditions in the targeted population are essential. This study explores and describes the views of drivers and conductors on the causes of WPV and ways of preventing it in the road passenger transport sector in Maputo City, Mozambique.

**Methods:**

The design was qualitative. Participants were purposefully selected from among transport workers identified as victims of WPV in an earlier quantitative study, and with six or more years of experience in the transport sector. Data were collected in semi-structured interviews. Seven open questions covered individual views on causes of WPV and its prevention, based on the interviewees' experiences of violence while on duty. Thirty-two transport professionals were interviewed. The data were analyzed by means of qualitative content analysis.

**Results:**

The triggers and causes of violence included fare evasion, disputes over revenue owing to owners, alcohol abuse, overcrowded vehicles, and unfair competition for passengers. Failures to meet passenger expectations, e.g. by-passing parts of a bus route or missing stops, were also important. There was disrespect on the part of transport workers, e.g. being rude to passengers and jumping of queues at taxi ranks, and there were also robberies. Proposals for prevention included: training for workers on conflict resolution, and for employers on passenger-transport administration; and, promoting learning among passengers and workers on how to behave when traveling collectively. Regarding control and supervision, there were expressed needs for the recording of mileage, and for the sanctioning of workers who transgress queuing rules at taxi ranks. The police or supervisors should prevent drunken passengers from getting into vehicles, and drivers should refuse to go to dangerous, secluded neighborhoods. Finally, there is a need for an institution to judge alleged cases of employees not handing over demanded revenues to their employer.

**Conclusions:**

The causes of WPV lie in problems regarding money, behavior, environment, organization and crime. Suggestions for prevention include education, control to avoid critical situations, and a judicial system to assess malpractices. Further research in the road passenger transport sector in Maputo City, Mozambique and similar settings is warranted.

## Background

Violence occurs in all occupational sectors [1]. Workplace violence (WPV) is defined as "behaviors by an individual or individuals, within or outside an organization, that is intended to physically and/or psychologically harm a worker/workers and occurs in a work related context" [2].

The transport sector comprises air, rail, maritime and road transport [3]. The road transport sector is divided into cargo transport (also known as freight) and passenger transport [3]. In a European survey, the prevalence of physical and/or psychological violence over a 12-month period inflicted upon workers in road and pipeline transport has been found to be 11.5% [4]. In the USA, workers in the road and ground passenger sector accounted for 1.2% of all occupational homicides [5]. Studies of taxi drivers in Europe, Australia and the USA have estimated the prevalence of physical and/or psychological violence over 12 months at between 19 and 70% [6-8]. In Africa, a study in Mozambique reported prevalence of physical and/or verbal violence over the previous 12 months among taxi drivers at 48.6%, minibus drivers at 61.8%, bus drivers at 70.8%, and minibus and bus conductors at 66.0 and 75.0%, respectively [9]. The present study specifically addresses workers' views on WPV in the road passenger transport sector in Maputo City, Mozambique. Mozambique is a low-income country, located in south-east Africa, with a population of over 22 million [10]. It has an infant mortality rate of 118/1,000 births, life expectancy at birth of 52 years, a total adult illiteracy rate of 54.4%, and GDP per capita of 454 USD [10].

It is recognized that the interactions between contextual, individual, workplace and societal risk factors increase workers' vulnerability and exposure to WPV [1]. In the transport sector, the reported risk factors are: contextual, e.g. working in high crime areas [1,7]; individual-related, e.g. perpetrators of violence using alcohol or drugs, fare evasion [11,12]; victim-related, e.g. a failure to meet passengers' expectations [11,12]; workplace/environmental, e.g. overcrowded premises, working alone, working with the public, handling money and other valuables [6-8,12]; and societal, e.g. a violent society [1,13-16].

WPV has consequences for employers, such as increased absenteeism and financial loss [1,17], and also for workers, who experience emotional reactions, such as anger, fear, helplessness, sadness, and frustration, which result in poor performance and decreased job satisfaction [17-19]. Further, WPV has significant health impacts, including physical injuries, disabilities, death, psychosomatic complaints, emotional exhaustion, sleeplessness, anxiety, depression, post-traumatic stress, and low quality of life [20-26].

Studies from Mozambique have reported that WPV in the road passenger transport is a common phenomenon, occurring wherever workers are on duty, which suggests that employment in the sector may constitute an occupational and health hazard [9,26]. It is also reported that WPV is associated with burnout, although social support appears to buffer the effect of WPV on burnout [27].

The present study explores and describes drivers' and conductors' views on the causes of WPV in the road passenger transport sector in Maputo City, Mozambique, and on ways of preventing it.

## Methods

### The study setting

The study was carried out in Maputo, the capital city of Mozambique. The city has a population of over a million [10]. When data were collected (December 2009-March 2010), road passenger transport was provided in buses, minibuses and taxis, owned by one government company and the members of two private transport associations. Each bus and minibus was staffed by a driver and a conductor, while each taxi employed just one driver. The drivers and conductors are registered at Mozambique's National Traffic Institute (NTI), which is the government institution responsible for traffic control and regulation. The total population of registered drivers and conductors in the city in 2007 was 2,618, with the following distribution: 405 bus drivers, 377 bus conductors, 743 minibus drivers, 743 minibus conductors, and 350 taxi drivers. There is a male predominance among registered drivers and conductors.

Workers from the government company only drive and collect fares, while workers from the private associations have multiple duties. The conductors collect fares from passengers and are expected to assist the drivers in maintaining harmony among passengers. In addition, both the drivers and conductors help to load passengers' belongings and perform duties to ensure safety and hygiene in the vehicles. These duties include cleaning the vehicle, checking for mechanical problems, fixing punctures, and repairing any damage incurred to the vehicle. A driver or a conductor may also have a supervisory role, which includes resolving internal problems (between employees), and external problems (between employees and passengers). They also have to ensure that the vehicles have a valid license and undergo a yearly service, and that they follow the rules for orderly arrival at pick-up stations (e.g. taxi ranks, and bus or minibus terminals).

### Study design and participants

The study had a qualitative design, which made it possible to obtain an emic perspective i.e. the insiders' views [28]. Our interest was in capturing information based on the individuals' views on and experiences of WPV; the victimized individuals were identified in an earlier study [9]. These individuals' views can be understood as resulting from their experiences and how they relate to actual contextual conditions. The earlier study indicated that the highest percentage of victims of WPV was among drivers and conductors with six or more years of work experience [9]. Accordingly, eligible participants for the present study were the drivers and conductors who had been previously identified as victims of WPV, and who had six or more years of work experience. For the interviews, 38 workers were purposively selected to cover a variety of transport routes, taxi ranks, and bus or minibus drivers and conductors from different vehicles.

### Data collection

A letter was sent to the Chairman of the Commission Board of the NTI, requesting support in introducing the study to the chairmen of the private transport associations and the chairman of the government company. A meeting with the chairmen, deputy chairmen and general secretaries of the transport associations and the manpower director of the government company was arranged to obtain permission for the interviews to be conducted.

A semi-structured interview guide was constructed to explore the views of drivers and conductors on the causes of WPV and its prevention [28,29]. The questions focused on the workers' views, based on their experiences of being verbally threatened or abused, assaulted, bitten, slapped, hit, pushed, spat at, scratched, pinched, punched or kicked while on duty. A preliminary pilot interview study was conducted to clarify understandings of seven open-ended questions. The drivers and conductors were contacted face-to-face by the first author (MTC) at the bus terminals or taxi ranks. Each interview occurred soon after initial contact was made. Six contacted participants were not interviewed because they did not want their interview to be tape-recorded. The interviews were conducted in Portuguese, the official language of Mozambique, and each took about 30-45 minutes. Saturation, meaning that new information was no longer emerging, was considered to have been reached after 32 interviews [28]. The 32 transport workers interviewed were 8 taxi drivers, 6 bus drivers, 6 minibus drivers, 6 bus conductors, and 6 minibus conductors. In terms of socio-demographic characteristics, all the transport workers were males aged between 30 and 60; 14 had attended primary school (< 5 years in school), and 18 secondary school (10 to 12 years in school), and their occupational tenure in the transport sector ranged between 7 and 16 years.

### Data analysis

The interviews were transcribed verbatim from the tape-recorded material by the first author (MTC). Qualitative content analysis was used to explore the causes of WPV and suggestions for its prevention [30]. To establish familiarity with interview content, the entire collective text was carefully read through several times by the two Portuguese speaking authors (MTC and MS). The next step was to identify units of meaning concerning causes of WPV and suggestions for prevention. At a third step, the meaning units were condensed and coded, and then grouped into a matrix, based on their similarities, into different descriptive categories. Identification of codes and categories was first performed independently by MTC and MS, and then jointly verified. Then, the categories were allocated to themes in accordance with causes of WPV and suggestions for prevention, and then translated into English. Finally, the three authors together critically reviewed the analysis and approved its outcome. Examples of the matrix analysis are shown in tables 1 and 2.

**Table 1:** Examples from the content analysis of causes of workplace violence

Meaning unit	Condensation	Code	Category	Theme
*1-MMB5*. When the passenger doesn't want to pay and the conductor demands payment, there is a quarrel, with insults and threats from both sides	Does not want to pay, insults, threats	Does not want to pay	Managing money while transporting passengers	Money

*2-MMB6*. There are passengers who come onto the vehicle drunk ... these passengers provoke lots of problems, they insult, shove the conductor. When I stop for him to leave the vehicle, other passengers get angry because I'm causing a delay, so they insult the driver and the conductor	Drunken passengers, verbal abuse, shoving	Drunken passengers	Alcohol abuse	Behavior

**Table 2:** Examples from the content analysis of suggestions to prevent workplace violence

Meaning unit	Condensation	Code	Category	Theme
1-MA5. To prevent insults and threats from passengers, there must be education for passengers, and for drivers and conductors. There are lots of mass media, TV, radio, magazines; or a pamphlet could be written to spread information about how to behave in a chapa^1)^	Education for passengers, drivers and conductors, mass media distribution; write a pamphlet with information on how to behave in a chapa	Education for passengers and workers (radio, magazines, TV); spread information; write a pamphlet.	Practical education	Education

*2-MA1*. To prevent fights between colleagues we should have somebody to control our cars. The supervisors must be good at checking our cars at the bus terminals, e.g. by ensuring that two cars don't go out at the same time.	Vehicle control at bus terminals; supervisors must be efficient	At the bus terminals, vehicles must depart one at a time; supervisors must be efficient.	Departure of vehicles	Control

Chapa^1) ^= bus or minibus.

### Ethical considerations

The nature and aims of the study were explained to each participant and verbal consent was obtained. The participants were assured of confidentiality and informed of their right to withdraw from the interview at any time. Permission to tape-record the interview was sought from each participant prior to the interview, which was conducted at the convenience of the participant inside his vehicle. The National Committee of Bioethics for Health in Mozambique approved the study's methods and procedures.

## Results

The results of the analysis of themes related to the causes of WPV are presented first, followed by those for the proposals for prevention.

### 

#### Causes of Workplace Violence

The problems that constituted causes of WPV were subsumed under the following main themes: Money, Behavior, Environment, Organization and Crime.

### Money

#### Managing money while transporting passengers

Passengers not wanting to pay their fares, not having enough money or not agreeing with the fare, and also a lack of change, were regarded as triggers of violence from passengers against workers. A minibus driver said: *"When the passenger doesn't want to pay and the conductor demands payment, there is a quarrel, with insults and threats from both sides" *(MMB5). And a taxi driver stated: *"In my opinion, threats and insults at work are due to disputes over fares. When the driver tells the passenger the fare, he says no, you are robbing me, and then he starts to threaten you" *(MTC/S6).

#### Managing revenue owing to owners

A triggering circumstance that incited violence from the owners of vehicles against the workers was a failure to hand over the revenue demanded by the owners; workers either embezzled the money, although sometimes they asked for a salary payment on a fixed date. A taxi driver said: "*There are several reasons for violence from the owners of vehicles against the drivers; there are drivers who are not honest with their boss; they take all the money, or spoil the car, and the boss then insults or beats them" *(MTC/S2).

Further, drivers of buses or minibuses thought that accusing the conductor of embezzling money was a trigger of violence between co-workers. One bus driver said: *"... I say to the conductor we had lots of passengers, and the money doesn't match, you took the money. Then, there is a quarrel, up to the point that the driver assaults the conductor, or the conductor the driver. This is what causes fights, between co-workers, between drivers and conductors" *(MA4).

### Behavior

#### Alcohol abuse

Drivers and conductors commonly expressed the view that alcohol abuse caused a lot of violence from passengers against workers. An example comes from one minibus driver: *"There are passengers who come onto the vehicle drunk ... these passengers provoke lots of problems, they insult, shove the conductor. When I stop for him to leave the vehicle, other passengers get angry because I'm causing a delay, so they insult the driver and the conductor" *(MMB6).

Alcohol abuse by workers incited violence from the owners of vehicles against them, and there was also violence between co-workers, particularly from conductors against drivers.

#### Disdain, disrespect and disagreement

Participants from all occupational groups regarded disdain for their activities as a cause of violence from passengers against workers. A minibus driver put it as follows: "*In my view, passengers are pretty contemptuous of the work of chapa drivers*" (MMB1).

Disdain was also a source of conflict between co-workers. One conductor said: *"There are drivers and conductors who show no respect for each other, rather than working together. They talk about how you're dressed, or about your shoes, in a way that gets you annoyed and feel insulted*" (CMB6).

Rudeness due to disrespect was a trigger of violence from passengers against workers, between co-workers, and also from owners of vehicles against workers (and vice-versa). One minibus conductor said: *"Sometimes, conductors or drivers are rude to passengers, who then get angry and threatening" *(CMB4). Another reflected: *"There is violence between conductors and drivers because both are rude to each other; they lose their tempers and end up threatening each other" *(CMB2).

Disrespect for traffic rules, by breaching priority arrangements, was viewed by many workers as a trigger of violence, particularly between the drivers of buses and minibuses. One bus driver said*: "Breaking the priority rules often causes quarrels and sometime even fights between drivers at the bus terminal*" (MA5). Specifically, taxi drivers mentioned that disrespect for taxi-rank rules was a trigger of violence. One driver expressed it as follows: *"Threats and shoving arise from disrespect for the queue at the taxi rank; on some days, one driver has no customers while another takes them all. That causes a lot of trouble" *(MT7).

Disagreement between co-workers on the same vehicle was also a trigger of violence. This was described by one of the interviewed minibus drivers: "*Violence between conductors and driver does happen, e.g. when my conductor is rude to a passenger, and gets angry and insulting when I reprimand him" *(MMB1).

#### Failure to meet passengers' expectations and interference

By-passing a bus stop, thereby shortening the transport route, not stopping at a bus stop, and transport delays, especially when passengers are in a hurry, were viewed as triggers of violence from passengers against workers. One bus conductor said: *"When a bus stop is missed, the conductor is shoved, or the driver insulted or threatened" *(CA6). Another worker explained: *"Because of many traffic-police stops or traffic jams, the bus takes longer to reach the terminal, so the passengers react as well" *(MA1).

Drivers sometimes viewed conflict mediation between passengers and co-workers as a form of interference and trigger of violence from passengers against workers. One minibus driver said: *"For example, a passenger assaults or insults the conductor, and when the driver intervenes, he too is insulted or assaulted by that passenger" *(MMB1).

### Environment

#### Overcrowding, breakdown and damage

The workers on buses and minibuses expressed the view that an overcrowded vehicle was a trigger of violence from passengers against workers. One example comes from a bus driver: *"The passengers start to argue when the bus is overcrowded, but the conductor continues to let more passengers on. If somebody steps on or shoves another one, the passenger gets furious, and insults the conductor and the driver" *(MA3). Insisting that passengers stand back also triggers violence. A minibus conductor expressed this as follows: *"What incites passengers to threaten, to shove, to insult a conductor is when the bus is already overcrowded and more passengers are allowed to get on ... I ask a passenger to stand back but he doesn't want to, and if I insist, then there is a dispute*" (CMB5).

Vehicle breakdowns and accidents were triggers of violence from owners of the vehicles against their workers. A minibus conductor explained: *"The bosses get furious, and threatening or insulting when the car breaks down, or when there is an accident, even when it's not the fault of the driver or conductor*" (MMB3). This also happens when the vehicle is not looked after, e.g. dirty or scratched. An example comes from a minibus conductor: *"If the car isn't cleaned or washed, the conductor is insulted by his boss*" (CMB4). And a taxi driver said: *"The owner of the vehicle threatens the driver if he doesn't look after the car, or if it gets scratched due to careless driving*" (MT7).

Damage to passengers' belongings incited violence from passengers against workers. A minibus conductor said: *"The passenger got violent because his trousers were torn on the seat that the conductor offered him, and the two of them lost their tempers while trying to resolve the matter" *(CMB2).

### Organization

#### Fighting for passengers, rotas and multiple duties

Drivers of buses or minibuses blocking each other's way so as to get passengers first was a trigger of violence between co-workers. An example of this was given by a bus driver: *"A struggle starts because a driver won't give way so he can take the first passengers. When we return to the bus terminal there is a fight, with threatening behavior and assault*" (MA1).

Violence also occurred between conductors when one conductor took a passenger who was first called by another. A minibus conductor stated: *"The struggle to get passengers happens mostly at bus stops; when a conductor takes a passenger who was first called by another, there are threats, insults and shoving" *(CM2).

One taxi driver stated that some drivers being given more days on the duty rota was a trigger of violence between co-workers. He explained: *"... but the duty rota doesn't follow any rules; there are drivers who get more days, which is what provokes the fighting" *(MT4).

Also, conductors with multiple duties were recipients of violence from owners of the vehicles. A minibus conductor said: *"There is violence due to lack of car inspection and control. Car control includes car cleaning, changing oil, checking whether the drivers are driving carefully, changing tires, and checking mechanical problems. The conductor's job is to collect money from passengers, but when he is not able to do all these other things, he starts fighting with the boss" *(MMB2).

### Crime

Taxi drivers pointed to robbery as a cause of violence. This was described by one of the drivers as follows: "*I take people who ask to be taken to secluded neighborhoods where there isn't much traffic, but where there are rarely any police. On arrival, the taxi driver gets threatened by two or three men, and he can't do anything about it; they beat him up and take all his money and other possessions*" (MTC/S1). There is a lack of security for taxi drivers, which causes violence. Another said: "*We carry all sorts of passengers, sick people, bandits, criminals; we don't know who they are, and then we get hurt*" (MT3).

#### Suggestions for WPV Prevention

Suggestions for prevention were subsumed under four main themes: Education, Control, Conflict Avoidance and Institutional Judgment. Table 3 shows the suggestions for WPV preventive interventions made by the participants.

**Table 3:** Suggestions for workplace violence prevention

Education	Control	Conflict Avoidance	Institutional Judgment
*Formal education* Train employees in conflict resolution, and employers in passenger transport administration *Practical education* Employers to get to know the job of transport workers by staying in the vehicle for some time Passengers to be informed when fares are raised by using the media or pamphlets. *Moral education*, Promote moral considerations with regard to traveling collectively	*Conflicts with passengers regarding money* Inspectors to be more efficient in controlling fare evasion Employers to control workers' activities by registering mileage when handing over the vehicle Have supervisors in the transport sector. *Organizational problems between co-worker* Supervisors to be more efficient in controlling the departure of vehicles from bus terminals	A) *Managing specific situations* Due to money problems, e.g. embezzlement of revenue owing to owners, increase the salaries of conductors, and pay the salaries on a fixed day each month Due to environmental problems, e.g. vehicle breakdown because of careless driving, the drivers should pay for the spare vehicle parts. Stop shortcutting of transport routes Traffic police and inspectors to be more efficient in dealing with vehicle overcrowding Due to organizational problems, e.g. the straggle to get passengers, have timetables for buses/minibuses and organized bus terminals Avoid multiple duties for workers Workers on buses/minibuses who do not respect queuing rules should incur penalties from supervisors and fines by the police for priority breaches *B) Managing specific behaviors* Workers should be banned for some days, and expelled if their bad behavior continues. If passengers lose their temper or get abusive, workers should not react, but remain calm and silent. When a drunken passenger wants to get into a vehicle, the police or supervisor must be called to intervene Fine the perpetrator in cases of violence between co-workers *C) Identifying passengers* Avoid passengers who are drunk, and avoid taking passengers to secluded neighborhoods	Create an institution to adjudicate between employers and workers

### Education

It was suggested that formal, practical and moral education might prevent WPV resulting from money, organizational and behavioral problems. Proposals for formal education of workers and owners of vehicles included getting workers to attend training courses organized by the transport associations, and the owners of vehicles going on courses about the administration and management of passenger transport. A minibus driver said: *"I think it has to do with education, because if drivers and conductors are properly trained, they can resolve the conflicts. Training is essential" *(MMBS/2).

Several suggestions for the practical education of passengers, workers and owners of vehicles emerged from the interviews. It was proposed that the transport associations or owners of the vehicles should have a duty to inform the community when fares were about to rise, and also to use the media to show how to behave on public transport. One example was given by a bus driver: *"To prevent insults and threats from passengers, there must be education for passengers, and for drivers and conductors. There are lots of mass media, TV, radio, magazines; or a pamphlet could be written to spread information about how to behave in a chapa" *(MA5).

Another suggestion was that the owners of vehicles should stay in a vehicle for some time, getting to know what the job of transport worker is like. A bus conductor said: "*There are days with no activity or with traffic jams; the boss should be in a car for a week or so, then he would get to know what chapa work is like" *(CA5).

There were also proposals for the moral education of passengers and workers that included learning how to behave well in the transport sector and how to respect the rules. For example, in the case of passengers, the transport associations should sensitize the community in general by spreading documents about how to behave while traveling. One bus driver made the following proposal: "*To prevent insults, assault and threats from passengers, the community in general should be made to know that when people are traveling collectively, they must respect other passengers, and also the conductor and the driver ... The transport associations can use documents to sensitize citizens about traveling behavior*" (MA4). In the case of workers, there should be weekly or monthly meetings with counselors to learn how to behave at work. One bus driver explained this suggestion: *"... A weekly or monthly meeting for drivers to talk about how to do the work might give us an organization; now we're disorganized; we need a counseling center to get to know how we should behave at work" *(MA3).

### Control

A suggestion for preventing WPV due to money and organizational problems was to improve control. In particular, with regard to money conflicts with passengers, inspectors should be more efficient in controlling fare evasion. One example was given by a minibus driver: "*An inspector knows how to handle a passenger who does not want to pay" *(MMB5).

And the owners of vehicles or transport associations should control workers' activities by registering mileage when handing over the vehicle, having supervisors, and finding out if there are issues involving the heads of taxi ranks. A taxi driver suggested: *"To prevent violence between boss and driver, the boss must tell his driver what he wants and check on his daily activities, like recording the mileage when he hands over the car" *(MTC/S6).

Regarding organizational problems between co-workers, a suggestion was that supervisors should be more efficient in controlling the departure of vehicles at bus terminals. The view of one bus driver was: "*To prevent fights between colleagues we should have somebody to control our cars. The supervisors must be good at checking our cars at the bus terminals, e.g. by ensuring that two cars don't go out at the same time*." (MA1).

### Conflict Avoidance

#### Managing specific situations

To prevent WPV due to money problems, such as the embezzlement of revenue due to the owners, suggestions included increasing the salaries of conductors and paying workers on a fixed day in the month. A bus conductor said: "*The bosses must improve the salary of the conductors; we earn 2000 MTs *(about 63 USD) a month, *the drivers 3000 MTs *(about 94 USD). *Increasing our salary would prevent the withholding of revenue money*" (CA1).

For preventing WPV due to environmental problems, such as a breakdown due to careless driving, one suggestion was to apply sanctions to workers, e.g. by making drivers pay for spare car parts. One of the bus conductors said: "*The boss must establish whether the breakdown of the car was due to careless driving on the part of the driver, and then apply sanctions, e.g. make the driver pay for the spare parts if he is responsible. This must be agreed when the driver starts employment*" (CMB2).

Other environmental problems, such as overcrowded vehicles and shortcutting transport routes, might be avoided by getting the traffic police and inspectors to be more efficient. A bus driver said: *"The vehicles must be in good condition and not overcrowded, and the routes must be respected. To achieve this, the police and inspectors must be more efficient" *(MAS6).

Regarding organizational problems, e.g. the struggle between transport workers to get passengers, suggestions for preventing WPV included having timetables for buses and minibuses, and properly organized bus terminals with defined ways in and out for the vehicles. A bus conductor said*: "When the chapa has a timetable, it puts an end to the fight to fill up the car" *(CA1). And, as a minibus conductor put it: "*A sizeable terminal, well organized, with ways in and out of the car queue ... would stop violence due to the getting of passengers" *(CMB2).

It was claimed that WPV due to organizational problems, such as workers having multiple duties, could be prevented by vehicle owners employing more workers. A minibus conductor said: *"... one driver and one conductor can't perform all the duties, clean the car, collect the money and repair the tires ... the bosses must employ more workers" *(CMB1).

To prevent WPV because of crime against taxi drivers, one proposal was that the drivers should carry firearms. One taxi driver said: *"We must ... even have a gun, so the bandits who pretend to be passengers and afterwards threaten, beat and steal from you, will know that a taxi driver has a gun .... Then, they will be afraid to touch or provoke you; they will know you might have a shotgun" *(MT3).

Finally, to prevent WPV due to behavior problems, such as not following the rules of the taxi rank, it was suggested that some workers should be banned from the rank for some days, and, if their bad behavior continued, then expelled. One taxi driver said: *"If a colleague doesn't follow the queuing rules, he should stay off the rank for some days as punishment" *(MTC/6). Also, the workers on buses and minibuses suggested surveillance of and penalties on drivers, applied by supervisors, who did not respect the queue, and fines by the police for breaches of priority rules. A bus driver suggested*: "If necessary, surveillance with a penalty should be applied to the ones who don't follow the queue at bus terminals, and the police must impose fines when there are breaches of priority. Surveillance must work; otherwise, there will always be problems" *(MA5).

#### Managing specific behaviors

Other suggestions for preventing WPV due to behavior problems were to avoid or manage specific behaviors. For example, when a passenger starts to abuse or lose his temper, workers should not react, but remain calm and silent instead. When drunken passengers want to get into a vehicle, a supervisor or the police should be called to intervene. Finally, fines were proposed for transport workers who incite violence. One minibus driver said: "*Drunken passengers who want to get on the bus must be prevented by supervisors or the police" *(MMB6). Regarding violence from a co-worker, the proposal was for fining the perpetrator. A minibus conductor said: *"I suggest fining anyone who starts to incite violence*" (CMB2).

#### Identifying specific passengers

To prevent WPV due to behavior problems, one suggestion was to identify potentially troublesome passengers, e.g. the ones who are drunk, and avoid carrying them. A taxi driver put it as follows: *"If it appears to me that someone is drunk, I try to avoid letting him into the car ... I try to avoid the problem by saying that the car isn't equipped to do the trip he wants" *(MTC/S1).

Another suggestion for preventing crime-related WPV was not to go to secluded neighborhoods, saying that the vehicle does not meet the conditions for the trip. One taxi driver explained: *"... if passengers appear ... two or three men who want to go to a remote neighborhood, I avoid carrying them. I do it by saying that the car does not meet the conditions to perform the trip they want" *(MTC/S1).

### Institutionalized judgment

In addition to other suggestions for preventing WPV from owners of vehicles or associations against workers because of money problem, it was suggested that there should be an institution to judge vehicle owners and workers when demanded revenue was not handed over. A driver of a minibus proposed: "*To stop vehicle owners from slapping and delivering insults when we don't hand over the amount of daily revenue they expect, we must have a place to which the vehicle owner, driver and conductor can refer, and a judgment made over who is right; the one who is wrong should incur a penalty" *(MMB6).

## Discussion

The present study provides important knowledge about workers' views on causes of WPV in the road passenger transport sector in Maputo City, Mozambique. The main causes and triggers described have similarities with findings reported in earlier studies. They include individual risk factors on the part of perpetrators (alcohol abuse, fare evasion) [11], individual risk factors on the part of victims (failure to meet passengers' expectations) [11,12]; and workplace-specific risk factors (environmental, e.g. overcrowded vehicles; task situation, e.g. managing money) [6-8,11]; societal risk factors (e.g. crime, especially robbery) [13-16]. However, differences were found between the findings of the present study and those of others with regard to workplace risk factors and organizational problems. One problem was the struggle faced by transport workers in Mozambique to get passengers. In a study of railway workers and air crew in the UK, a substantial problem lay in disputes over baggage [11]. An explanation for such a difference is related to the different work conditions that apply in a low-income country like Mozambique, and in a country like the UK with welfare provisions and high income. In Mozambique, the unemployment rate in 2010 was 18.6% [31]. The monthly salaries of drivers and conductors were equivalent to 94 USD and 63 USD, respectively. In Maputo City, public transport is largely provided by the buses and minibuses of private associations. Passenger transport is a money-making business for the owners of the vehicles and the associations involved. Also, the transport workers are dependent on employment by the vehicle owners for their own survival and that of their families. Thus, drivers and conductors are engaged in a highly competitive struggle for passengers.

Other findings concerning the main triggers and causes of violence in the present study were: revenue demanded by vehicle owners not being handed over, money being embezzled, breaching of queuing rules at taxi ranks, and taking short cuts on prescribed transport routes. These findings also seem to reflect problems related to low income. No such comparable findings have been reported in earlier studies conducted in high-income countries in Europe, in the USA, and in Australia [6-8,11,12].

Further, the workers provided some insights into ways of preventing WPV in their own occupational settings. Their suggestions for the prevention of WPV are comparable to those found in earlier studies, which include having police and supervisors control fare evasion, intervention by the police to stop drunken passengers getting into vehicles, and the provision of education [3,12,32]. But, the findings of the present study also reveal a lack of comparability with earlier studies, which may be understood in terms of the different work conditions and contextual conditions that apply in a low-income society. For example, the buses and minibuses of the private associations in Maputo did not have timetables, organized bus terminals or pre-paid fares.

And there were further divergences from earlier studies with regard to the kinds of education suggested by the interviewees. The workers in the present study proposed practical education for employers (owners of vehicles), e.g. by being in the vehicles sometimes to understand what the jobs of drivers and conductors involve. Moral education was proposed in order to teach both passengers and workers how to behave properly during personal transportation. In earlier studies [1,3,12,32], neither practical nor moral education was proposed as a means for reducing violence from passengers against workers and among co-workers. The behaviors concerned, which involve honesty, responsibility and respect for others, lie within the moral domain [33-35]. The idea that passengers in Maputo City are violent because of the moral issues that arise while using public transport needs to be questioned. It seems more credible that there are some conditional considerations that outweigh passengers' and workers' moral virtues. The interviewees' suggestions for moral education do not fully address the question of which conditions influence their moral stances. It is a limitation of the present study that the open-ended questionnaire employed did not permit follow up of this issue. However, the interviewees' proposals for using TV, radio, magazines or targeted pamphlets to spread information on good behavior while traveling collectively may lead to the promotion of awareness in the community about moral issues.

Other proposals for preventing WPV, suggested in the present study, that were not found in earlier studies included: having an institutional arrangement to judge employers and workers when demanded revenue is not handed over; drivers having to pay for spare vehicle parts when breakdowns are confirmed to have been due to careless driving; and, imposing fines on workers who incite violence. However, implementing the proposal that taxi drivers should carry a firearm as a way of preventing crime in adverse situations would probably increase rather than reduce the risk of violence [36,37].

### Implications for prevention and research

Intervention programs usually rest on prior understanding of the context and knowledge of the conditions in the targeted population [38]. Interventions to prevent WPV in the transport sector should target not only workers and employers but also members of the community. This can be achieved by providing formal education, e.g. training workers in conflict management, and employers in passenger transport administration, and - at community level - improving work conditions at the terminals [1,3,39-41].

In Maputo City, Mozambique, before preparing a preventive intervention, additional groups need to be approached, such as owners of vehicles and associations, members of the community, supervisors, and the traffic police. The proposed strategies have to be discussed.

Studies of WPV in the transport sector are generally scarce, and few have been conducted in low-income countries. Further research into the causes of WPV and its prevention in the road passenger transport sectors in Maputo, and also in similar settings, is warranted. It would increase knowledge about WPV, and might help to initiate appropriate preventive interventions.

### Methodological strengths and limitations

This study was planned, analyzed and finalized by the three co-authors, each with a different professional background, which has strengthened the study design. Accuracy, credibility, transferability, dependability and conformability are the commonly employed criteria for assessing trustworthiness in qualitative research [29]. Regarding dependability and conformability, while conducting the interviews, the first author's priority was to maintain a neutral stance, despite prior understanding of work conditions in Maputo, by trying not to make any judgments about the information provided or by suggesting solutions to problems. The views expressed by drivers and conductors are based on their individual experiences as victims of WPV in the road passenger transport sector. To improve accuracy, the information from the drivers and conductors was nuanced by two Portuguese-speaking researchers before the constructed themes were translated into English. Credibility was also improved by the validation of the second author. The transferability of the findings in this study to other road passenger settings is restricted to other low-income countries with similar transport conditions.

Reasons were not given why six potential participants among the drivers and conductors did not want to be interviewed when they heard that their views were to be tape-recorded. One explanation might be that they found the revealing of causes of violence too sensitive, or they might even have been perpetrators themselves. The non-participation of the six workers might have diminished information on the views of drivers and conductors on causes and means of prevention of WPV in the sector. However, the results are unlikely to have been affected, since the views expressed by the 32 workers are comparable with those found in previous studies [7-9,11-16].

## Conclusions

The causes of WPV in the road passenger transport sector in Maputo City, Mozambique included problems regarding money, behavior, environment, organization and crime. In order to prevent WPV in the sector, interventions should target workers and employers, and also members of the community. Moral, practical and formal kinds of education were proposed. Educational programs may give rise to some positive changes in the behaviors of passengers and workers, including knowing how to behave when traveling on public transport and following the rules. Educational programs can provide training to workers, e.g. in how to resolve conflicts between co-workers and with passengers, and to employers about the administration and management of passenger transport. In addition to education, there were proposals for control strategies, the avoidance of critical situations, and the introduction of some kind of judicial system.

Others groups need to be approached, such as the owners of vehicles and the transport associations, members of the community, supervisors and the traffic police, before preparing preventive interventions in Maputo City in Mozambique.

Our findings suggest the need for further research into the causes of WPV and its prevention in the road passenger transport sector in Maputo City, Mozambique, and in similar settings. This would increase knowledge of WPV and help to set up appropriate preventive strategies to reduce it.

## Competing interests

The authors declare that they have no competing interests.

## Authors' contributions

MTC has been central to the design and planning of the study. She conducted and transcribed the interviews, performed the analysis and conceptualization together with MS, and drafted the first manuscript. MS and PT contributed to the conception, planning and design of the study, were involved in the analysis, and also took part in the writing and finalization of the manuscript. All the authors read and approved the final manuscript.

## Pre-publication history

The pre-publication history for this paper can be accessed here:

http://www.biomedcentral.com/1471-2458/11/800/prepub
